# Diagnostic profile among patients with chronicurticaria/angioedema attending a reference clinic in Brazil: the role of auto-immunity

**DOI:** 10.1186/1939-4551-8-S1-A104

**Published:** 2015-04-08

**Authors:** Daniel Cordeiro, Priscila Palhas, Phelipe Souza, Karine Boufleur, Lucas Brom Thais Nociti, Janaina M Melo, L Karla Arruda

**Affiliations:** 1 Ribeirao Preto Medical School, University of Sao Paulo, Brazil

## Background

Management of patients with chronic urticaria/angioedema is often a challenge to the Allergist/Immunologist. We aimed to evaluate the diagnostic profile among patients with chronic urticaria/angioedema attending a reference clinic in Brazil.

## Methods

Two hundred and fifty four patients aged 13-80 years (202 females,79.5%), with chronic urticaria/angioedema who receive care at the Allergy Clinic of the Clinical Hospital of Ribeirão Preto Medical School in Brazil, were evaluated prospectively. Patients were inquired about triggering of symptoms by medications and/or physical agents and presence of anaphylaxis. In addition, they were investigated by skin testing and tests for eosinophilia, IgE, and hepatitis B and C. Autoimmune basis was characterized by presence of positive autologous test, anti-thyroid or antinuclear antibodies (ANA). When indicated, a skin biopsy was performed. Use of medications for urticaria was analyzed.

## Results

The disease had a duration ranging from 6 months to 36 years among the patients participating in the study. Sixty-six patients (26%) had urticaria only; 167(66%) urticaria and angioedema; and 21(8%) angioedema only. Of the 118 patients who underwent autologous skin testing, 56 (47.4%) showed positive results. Anti-thyroid antibodies and ANA were positive in 12% and 10.6% of patients, respectively. Eighty-five (33.5%) patients reported triggering of symptoms by medications, in particular by ASA/NSAIDs (16.5%). Thirty-two percent of patients reported worsening of symptoms by physical agents. Eight patients reported anaphylaxis. Biopsy carried was out in 23 patients, and the results revealed 4 with urticaria vasculitis, 5 with eosinophilic infiltrate and 14 with non-specific findings. Sixty percent of patients had total IgE>100kU/L; positive skin test were more frequent to mites (40%), cockroach (28%) and shellfish (13.3%). Ninety-two percent were in use of at least one daily medication, mostly anti-histamines (85%); 17/254 (7%) were in long term use of oral corticosteroids.

## Conclusions

Features of autoimmune urticaria/angioedema were identified in 90/254 patients evaluated (35.4%). Other causes included physical, food-induced, and NSAIDs-induced urticaria. Hereditary and ACE-inhibitor induced angioedema were diagnosed in 3 and 5 patients, respectively. Despite extensive investigation, 98 patients (38.5%) remained diagnosed as spontaneous urticaria/angioedema.

